# Expression of TRF2 and its prognostic relevance in advanced stage cervical cancer patients

**DOI:** 10.1186/0717-6287-47-61

**Published:** 2014-11-25

**Authors:** Sevgi Ozden, Pinar Mega Tiber, Zerrin Ozgen, Hazan Ozyurt, Nedime Serakinci, Oya Orun

**Affiliations:** Clinic of Radiation Oncology, Dr. Lutfi Kirdar Kartal Training and Research Hospital, Semsi Denizer Street, Istanbul, 34890 Turkey; Biophysics Department, Marmara University School of Medicine, Maltepe Basibuyuk Yolu Street, Istanbul, 34854 Turkey; Department of Radiation Oncology, Marmara University, School of Medicine, Muhsin Yazicioglu Street, Istanbul, 34890 Turkey; Near East University, Faculty of Medicine, Medical Genetics, 922022 Lefkosa, KKTC Mersin 10, Turkey

**Keywords:** Cervical cancer, Radiotherapy, Telomere repeat-binding factors 2, B-cell lymphoma-extra large, Apoptosis

## Abstract

**Background:**

Telomeres are protective caps consisted of specific tandem repeats (5′-TTAGGG-3′). Shortening of telomeres at each cell division is known as “mitotic clock” of the cells, which renders telomeres as important regulators of lifespan. TRF2 is one of the critical members of shelterin complex, which is a protein complex responsible from the preservation of cap structure, and loss or mutation of TRF2 results in DNA damage, senescence or apoptosis. Since cancer is frequently associated with aberrant cell cycle progression, defective DNA repair or apoptosis pathways, TRF2 could be one likely candidate for cancer therapy.

Here we investigated the prognostic role of TRF2 levels in cervical cancer patients. Fold-induction rates were evaluated with respect to median values after real-time PCR analysis. Overall survival, distant disease-free and local recurrence-free survival rates were calculated using Kaplan-Meier long rank test.

**Results:**

Both five year overall- and disease-free survival rates were longer in patients with higher TRF2 expression compared to lower expression, but results were not statistically significant (69.2% vs 28.9%, respectively). Mean local recurrence-free survivals (LRF) were very close ( 58.6, CI: 44.3-72.9 vs 54.5, CI: 32.1-76.9 months) for high and low expressions, respectively. Cumulative proportion of LRF at the end of five year period was 76.9% for high and 57.1% for low TRF2 expression (P = 0.75). Statistically significant difference was found between survival ratios and Bcl-xL and p53 gene expressions, but not with TRF2. A respectable correlation between TRF2 expression and apoptosis along with distant metastasis was noted (P = 0.045 and 0.036, respectively). Additionally, high TRF2 expression levels had a positive impact in five year survival rate of stage IIIB-IVA patients (P = 0.04).

**Conclusions:**

Our results support the role of TRF2 in apoptosis and imply a positive relation with distant metastases and survival in advanced stage patients. The remarkable difference in survival periods of patients with different TRF2 expressions suggest that TRF2 may be a candidate factor to estimate survival for cervical cancer, a preliminary observation which should further be verified with a larger cohort.

## Background

Telomeres are protective caps consisted of specific tandem repeats (5′-TTAGGG-3′). Shortening of telomeres at each cell division is known as “mitotic clock” of the cells, which renders telomeres as important regulators of lifespan. The cap structure of telomeres is preserved by shelterin complex consisted of six proteins, telomere repeat-binding factors 1 and 2 (TRF1, TRF2), protection of telomeres protein 1 (POT1), heterodimeric partner of POT1 known as TPP1, TRF1-interacting nuclear factor TIN2 and RAP1 [1, 2]. This structural organization serve to protect ends of chromosomes from end fusions or DNA repair proteins and participates to the genomic stability. TRF2 is one of the critical members of shelterin complex and loss or mutation of TRF2 results in DNA damage, senescence or apoptosis [3, 4]. Since cancer is frequently associated with aberrant cell cycle progression, defective DNA repair or apoptotic pathways, TRF2 could be one likely candidate for cancer therapy.

Cervical cancer is one of the most common malignant gynecological disorders, especially in less-developed countries. Even though this cancer type is mostly based on human papilloma virus infection, 5-year overall survival rates are still around 52% after application of combined radio-chemotherapy [5]. In HPV based type cervical cancers, integration of virus DNA into host’s DNA results in the overexpression of especially two viral oncogenes, E6 and E7, which deregulates both cell cycle and apoptosis in the cell. Activation of telomerase reverse transcriptase (TERT) by E6 is one of the crucial steps causing immortalization [6].

In addition to telomerase activation, infection by high-risk HPV types has been found to be associated with genomic instability [7]. Genomic instability is a well-known factor driving the cells to a malignant phenotype. There are wide varieties of factors contributing to genomic instability and giving rise to high heterogeneity among the tumor cells. Since TRF2 is an important element of telomere homeostasis and loss of TRF2 yields end-to-end fusions, telomere shortening, activation of DNA damage pathways, TRF2 regulation could be a contributing factor in cancer progression [4]. In accordance with this hypothesis, TRF2 overexpression is detected in various tumor types like gastric carcinoma, hepato-carcinogenesis or colorectal carcinoma [8–10]. Regulation of TRF2 expression was found to be correlated with tumor grade in lung cancer progression [11].

As human telomerase and the cap structure at telomere regions are important contributors of instability and cancer progression, we wanted to know potential role of TRF2 protein, an important protector of cap structure, on the prognosis of advanced stage cervical cancer patients. Gene expression of TRF2 was quantified by real-time PCR. Values were normalized relative to β-actin levels and compared to previously determined levels of apoptotic gene B-cell lymphoma-extra-large (Bcl-xL) and tumor suppressor p53. Bcl-xL is an anti-apoptotic protein and relative expression of anti- and pro-apoptotic proteins are the determinants of cells’ faith on survival. p53, on the other hand, is a well-known tumor suppressor, mutation of which was shown to lead uncontrolled cell proliferation.

In this study, Association between TRF2 expressions and survival rates of advanced stage cervical cancer patients has been investigated and correlations of TRF2 expression with above mentioned elements of cell survival and apoptosis were determined in this perspective.

## Results

TRF2 expression was determined in cervical cancer patients admitted to the Dr. Lutfi Kirdar Kartal Training and Research Hospital Radiation Oncology Department. The mean follow up period was 63.2 months (standard error of the mean (S.E.M) was 6.6). Histopathological evaluations of patients revealed that 40% has invasive and 60% has large cell squamous cell type. Expression levels were determined using real-time PCR analysis and values were normalized against house-keeping gene beta-actin. Fold-induction ratios were evaluated and assessed as “high” or “low” with respect to the average of the group. The mean from a small pool of normal cervical tissue was used as complementary reference. Median age of patients was 56 (range 39–75). Overall survival rates at the end of 5 year period were analyzed against clinical parameters of the patients (Table 1). Survival times were straightly correlated with the stage and grade of the disease as expected. In addition, grades were also strongly correlated with distant metastases (P = 0.001), while neither grade nor histopathological types found to be correlated with gene expressions. There was no apparent relation between survival and apoptosis.

**Table 1:** **Kaplan-Meier survival analysis for overall (OS) with respect to clinical parameters (n): number of patients**

	Mean survival (months)	(95% CI) (months)	OS 5 years (%)	P-value
**Stage (n)**				
**IIB (7)**	85.3	73.9-96.8	85.7	0.02*
**IIB-IVA(13)**	53.7	32.7-70.1	38.5	
**Age**				
**>56 years**	71.3	56.3-86.4	60	0.28
**<56 years**	57.8	37.1-78.4	50	
**Apoptosis (n)**				0.18
**High (9)**	70.7	52.7-88.6	63.6	
**Low (11)**	57.4	39.1-75.7	44.4	
**Grade (n)**				0.06
**High grade (11)**	53.6	36.6-70.6	33.3	
**Low and medium grade (9)**	81.0	66.5-95.4	87.5	

*Correlation is significant at the 0.05 level (2-tailed).

Immunohistochemical evaluation of apoptotic cells were determined by TUNEL staining as described in methods. Sections from the paraffin block firstly deparaffinized and proceeded according to kit’s instructions. Apoptotic nuclei were visualized using the DAB substrate, which resulted in brown precipitate.

**Figure 1:**
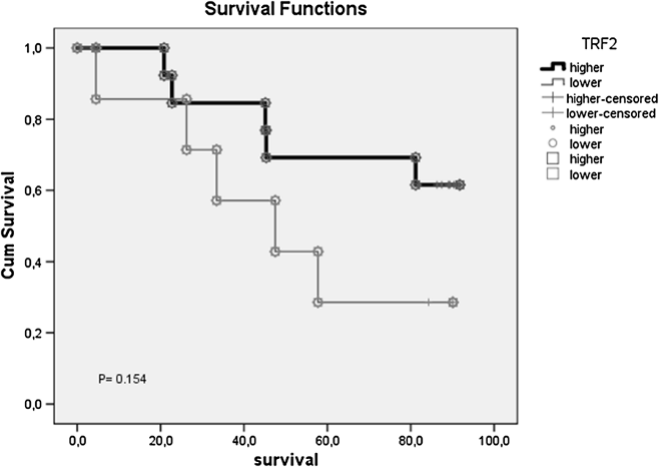
**Kaplan-Meier survival curves with univariate log-rank comparisons for 8 year follow up period for TRF2 expression.**

Mean survival was found as 65 months (S.E.M. 6.7) with 95% confidence interval (CI), 51.8-78.4. Mean overall survival time in patients with higher TRF2 expression than average was longer compared to lower expression levels, as 73.0 months (S.E.M. 7.5) vs 49.9 months (S.E.M. 11.2) with 95% CI, 58.1-87.8 and 27.9-71.9 respectively. Again, 5-year overall- and disease-free survival rates were longer in patients with higher TRF2 expression compared to lower expression (69.2% vs 28.6% for overall and the same for disease-free survivals, respectively), but results were not statistically significant (Table 2). The Kaplan-Meier survival curve of the log-rank analysis explicitly showed that low OS (overall survival) expression levels might well be related to relative low level TRF2 expression (Figure 1). Local control means were very close in both groups (58.6 months [95% CI, 44.3-72.9] vs 54.5 months [95% CI, 32.1- 76.9] for high and low expressions, respectively. Cumulative proportion of local recurrence free survival (LRF) surviving at the end of 5 year period was 76.9% for high and 57.1% for low TRF2 expression.

**Table 2:** **Kaplan-Meier survival analysis for overall (OS), disease-free (DFS) and local-recurrence free (LRF) survival relative to gene expressions (n): number of patients**

	OS means (months)	95% CI (months)	OS (5 years)%	p-value
**TRF2 (n)**				
**High expression (13)**	73.0	58.1 – 87.8	69.2	0.154
**Low expression (7)**	49.9	27.9 – 71.9	28.6	
**Bcl-xL (n)**				
**High expression (11)**	46.5	30.4-62.7	27.3	0.002*
**Low expression (9)**	86.8	77.7-95.9	88.9	
**p53 (n)**				
**High expression (9)**	48.6	29.3-67.9	33.3	0.02*
**Low expression (11)**	78.1	64.2-91.9	72.7	
	**DFS means (months)**	**95% CI (months)**	**DFS (5 years)%**	**p-value**
**TRF2 (n)**				
**High expression (13)**	70.3	53.3 – 87.3	69.2	0.124
**Low expression (7)**	43.9	21.1 – 66.8	28.6	
**Bcl-xL (n)**				
**High expression (11)**	41.1	23.9-58.4	27.3	0.003*
**Low expression (9)**	84.8	72.0-97.5	88.9	
**p53 (n)**				
**High expression (9)**	45.8	25.7-66	33.3	0.04*
**Low expression (11)**	73.4	55.4-91.2	72.7	
	**Local control means (months)**	**95% CI (months)**	**LRF (5 years)%**	**p-value**
**TRF2 (n)**				
**High expression (13)**	58.7	44.4-72.9	76.9	0.750
**Low expression (7)**	54.5	32.2-76.9	57.1	
**Bcl-xL (n)**				
**High expression (11)**	45.4	26.4-64.3	45.5	0.01*
**Low expression (9)**	71.6	66.0-77.0	88.9	
**p53 (n)**				
**High expression (9)**	49.5	28.4-70.7	55.6	0.10
**Low expression (11)**	43.4	30.7-76	72.7	

*Correlation is significant at the 0.05 level (2-tailed).

Anti-apoptotic protein Bcl-xL and tumor suppressor protein p53, on the other hand, were markedly related to the survival ratios. 88.9% of patients with lower Bcl-xL expression had better survival time at the end of 5-year period (P = 0.002). The corresponding value was 72.7% for p53 expression (P = 0.02), in concert with its role as tumor suppressor (Table 2).

Spearman correlation analyses involving genes of interest, namely Bcl-xL, p53 and TRF2, together with apoptosis and distant metastasis, were introduced in Table 3. TRF2 was found to be related with number of apoptotic cells determined by TUNEL method (P = 0.045). This value was 0.069 for Bcl-xL and 0.369 for p53 protein. Local recurrence and differentiation were not correlated with any gene expression involved in this study (not shown) whereas presence of distant metastasis was negatively correlated with TRF2 (P = 0.036) (Table 3). High expression of TRF2 also resulted in longer survival in high grade patients when compared to low TRF2 expression group with high grade (mean survival was 58 months, S.E.M. 11.3, vs 45 months, S.E.M. 11.8, respectively), but results were not statistically significant (P = 0.14). The comparison of five year survival rates with the expression levels of Bcl-xL, p53, together with TRF2, on the other hand, were in close association with the stage of the patients, as represented in Table 4.

**Table 3:** **Spearman correlations for TRF2, Bcl-xL, and p53, together distant metastasis (DM), local recurrence (LR), stage and apoptosis and differentiation (Diff)**

	TRF2	Bcl-xL	Apoptosis	p53	DM	LR	Stage	Diff
TRF2	1,000	-0,032	0,453	-0,179	-0.471	-0,300	0,216	-0,171
P-value	-	0,895	0,045*	0,450	0.036*	0,195	0,359	0,471
Bcl-xL	-0,032	1,000	0,414	0,212	0.328	0,522	-0,474	0,082
P-value	0,895	-	0,069	0,369	0.158	0,018*	0,035*	0,731
Apoptosis	0,453	0,414	1,000	0,192	0.082	0,189	-0,267	-0,082
P-value	0,045*	0,069	-	0,418	0.731	0,424	0,256	0,731
p53	-0,082	0,212	0,192	1,000	0.082	0,436	-0,267	-0,082
P-value	0,731	0,369	0,418	-	0.731	0,055	0,256	0,731
DM	-0.474	0.328	0.082	0.082	1.000	0,471	-0,391	0,667
P-value	0.036*	0.158	0.731	0.731	-	0,036*	0,088	0,001*
LR	-0,303	0,522	0,189	0,436	0,471	1,000	-0,371	0,235
P-value	0,195	0,018*	0,424	0,055	0,036*	-	0,107	0,317
Stage	0,216	-0,474	-0,267	-0,267	-0,391	-0,371	1,000	-0,361
P-value	0,359	0,035*	0,256	0,256	0,088	0,107	-	0,118
Diff	-0,171	0,082	-0,082	-0,082	0,667	0,235	-0,361	1,000
P-value	0,471	0,731	0,731	0,731	0,001*	0,317	0,118	-

*Correlation is significant at the 0.05 level (2-tailed).

**Table 4:** **Comparison of clinicopathological parameters against TRF2, Bcl-xL and p53 expressions**

	TRF2 expression (n) OS (5 year,%)		Bcl-xL expression (n) OS (5 year,%)		p53 expression (n) OS (5 year,%)	
Stage (n)	High	Low	High	Low	High	Low
IIB (7)	5	2	2	5	3	4
	n.d.	50	n.d.	80	66.7	n.d.
IIIB-IVA (13)	8	5	9	4	6	7
	50	20	11	n.d.	16.7	57.1
P log rank	0.04*		0,006*		0,01*	
**Histologic grade (n)**						
High grade (12)	7	5	7	5	5	7
	42	20	28**	80	40**	57.1
Low grade (8)	6	2	4	4	4	4
	83	50	75	n.d.	75	n.d.
P log rank	0,14		0,01*		0,01*	

*Correlation is significant at the 0.05 level (2-tailed); ** 3-year survival; n: number of patients; n.d.: not determined, all censored.

As a protector of chromosome ends, high TRF2 levels had a positive impact on survival. There was no correlation between TRF2 and p53 expressions though it is known that shortage of TRF2 induce Ataxia Telangiectasia Mutated kinase (ATM) and p53 mediated apoptosis by activating DNA damage signal mechanism [12].

## Discussion

We studied the expression of TRF2, a telomere-associated protein and Bcl-XL and p53 genes in tumor tissues of cervix cancer patients and observed considerable difference in patients’ response with respect to relative protein expressions. Comparisons of mRNA levels and their post-operative long-term follow-up results were reported in advanced stage cervix cancer patients. Our results reveal that 69% of patients who have high intrinsic TRF2 expression had better survival periods (2.4 fold higher than the group with lower expression). Relative TRF2 levels were similarly associated with relative numbers of apoptotic cells, with a significant pair correlation (P = 0.045).

As a multifactorial event, cancer progression utilizes multiple components, one of them being the chromosomal instability. Induction of chromosomal instability through an unknown mechanism may be an important element to estimate long-term toxic effects of chemo-radiotherapy, tumor resistance or side-effects of the therapy as part of the novel personalized approaches in therapeutic applications. Telomerase enzyme and shelterin complex are protectors of telomere structure and function, thereby also protector of genome, preventing activation of DNA repair mechanisms at chromosome ends, such as end-to-end fusions, non-homologous end joining (NHEJ) etc. [4].

Cervical cancers differ from other types of cancer in that tumorigenesis is often a result of viral infection. E6 and E7 oncoproteins of human papilloma virus (HPV), virus responsible from nearly 70% of all cervical cancers, interferes with the cell-cycle and apoptosis regulatory pathways, initiating malignant transformation. E6 binds to the tumor suppressor protein p53 and directs it to ubiquitin-mediated degradation and E7 acts on retinoblastoma regulated network. However, integration of HPV DNA is not obligatory for malignant transformation, therefore whether genomic instability is a cause or a result of infection remains controversial [13].

Characteristics of tumor such as grade, stage, tumor size or localization are important prognostic factors in cervical cancer, as in the other types. However, genetic prognostic factors influencing survival are not yet well established. We have previously determined the role of several apoptotic factors in this perspective and found that a relatively new anti-apoptotic and reactive-oxygen-species (ROS) scavenger protein, sensitive-to-apoptosis (SAG) gene product, has a positive impact on prognosis of colorectal patients [14].

TRF2 is a small protein that binds to telomeric repeat units and stabilizes the DNA t-loop structure at telomere regions. The clinical potential of telomeric proteins in the treatment of cancer was subject of research by several studies [15–18]. The altered expression of telomere capping factors, such as TRF2, could enhance telomeric damage, cause to an instability at chromosomal ends, telomerase dependent shortening of telomeres and undesirable activation of DNA repair mechanisms. TRF2 is crucial in cap structure stabilization, it is an active DNA damage response protein and furthermore it even regulates polymerase activity of DNA polymerase β on telomeric and non-telomeric DNA substrates [19–21]. The potential role of TRF2 expression in solid tumors has been demonstrated in several studies. Overexpression of TRF2 was reported in variety of carcinomas, such as breast carcinomas, gastric carcinomas, colorectal carcinoma cells [22, 23, 10]. The transcription levels of TRF2 was found to be higher in benign samples and decreased with increasing stage and disease progression in human breast cancer tissue samples [22].

It is known that inhibition of TRF2 expression initiate apoptosis in many cell types [12]. Inhibition of TRF2 induces immediate alteration in cap structure and activates ATM or p53 dependent response pathways [24] p53, on the other hand, is a mediator of mitochondrial apoptotic pathway, mobilizing several downstream targets. Therefore assessment of differential expressions for TRF2 and previously determined apoptotic and cell survival related genes (Bcl-xL, p53) could provide valuable informations about the relationship between apoptosis, chromosomal stability and CRT treatments.

Here, we aimed to determine relative expressions of telomeric factor TRF2 in cervical-cancer tissues and its possible relation to survival, apoptosis and response to CRT treatments. TRF2 was found to be correlated weakly to apoptosis, but not to the survival. Although apoptosis related to TRF2 levels, expressions of apoptotic proteins did not show any correlation with TRF2 expressions. This result is not surprising since TRF2 induced activation of apoptosis possibly occurs through initiating chromosomal instability rather than activation of mitochondrial apoptotic proteins. Statistical evaluations were limited due to the small population size available. Besides the difficulty to obtain normal tissue in advanced stage patients and to separate it from tumor has prevented straight comparison relative to the same source. Results of our study indicate that Bcl-xL and p53 are strong candidates to estimate survival and prognosis as expected. TRF2 has a positive impact on survival and well correlated with apoptosis and distant metastases. Establishment of TRF2’s effect on survival through apoptosis reguires further investigation with larger cohort.

## Conclusion

Our results show that TRF2 expression is correlated with apoptosis, but not with the apoptotic gene expressions. A clear discrepancy exists between the survival rates of patients presenting high or low TRF2 expressions, but the results were not statistically significant. The observed correlation between high TRF2 expression and longer life spans of advanced stage cervical cancer patients suggests that TRF2, an important factor in chromatin stability and survival, might be a candidate factor to estimate survival in cervical cancers.

## Methods

### Study population and therapy

Tissues were collected from patients diagnosed with cervical cancer at Dr. Lutfi Kirdar Kartal Training and Research Hospital. The patient samples were classified according to Federation of Gynecology and Obstetrics (FIGO) system between stages IIB to IVA. Tissue samples were collected from patients after the receipt of formal ethical approval from the appropriate ethical committees related to the institution. All patients provided written informed consent before undergoing diagnostic cervical biopsy.

Patients were treated with a combination of external beam radiation therapy and high-dose-rate (HDR) remote control after-loading intra-cavitary radiation therapy. External pelvic irradiation was administered with 6–15 MV photon X-rays, where intra-cavitary radiation therapy was administered with HDR - Curietron Cesium-137 sources. A total dose of 50 Gy was delivered in 25–28 fractions as five fractions per week; afterwards, 20 Gy was delivered as brachytherapy at point A in three fractions as one per week. A short 1–2 hour infusion of cisplatin (40 mg/m^2^/d, once in a week) was applied as concomitantly during external radiotherapy. Regular follow-up consisted of physical examinations with 3 months intervals during the first two years, every 6 months interval thereafter. Patients in whom signs of metastatic disease were present underwent further investigations. Disease relapse was defined as either local recurrence or the development of metastasis and was determined by clinical and radiographic studies with CT or MRI or by tissue biopsy invariably. Overall- , disease free and local recurrences free survival percentages were determined for five years period.

### Real-time polymerase chain reaction

Total RNA was extracted from biopsies and processed as described previously [14]. Briefly, one microgram of total RNA was reverse transcribed using Transcriptor High Fidelity cDNA Synthesis kit (Roche Applied Science, Manheim, Germany) according to the manufacturer’s guidelines. cDNA’s were diluted 10 times and used as a template in real-time PCR reactions. TRF2 primers used in the analysis were: forward (5′- CGG GAT CCA TGG CCG ACG TGG AAG-3′) and reverse (5′- CGA AGC TTT CAT TTG CCG ATT CTT TGG AC-3′). Other primers for apoptotic protein Bcl-xL and tumor suppressor p53 were as follows: Bcl-xL forward (5′-CCA GAA GGG ACT GAA TCG-3′) and reverse (5′-CCT TGT CTA CGC TTT CCA C-3′), p53 forward (5′-AGG CCT AAC TCA AGG AT-3′) and reverse (5′-CCC TTT TTG GAC TTC AGG TG-3′).

### Apoptosis –TUNEL assay

Apoptotic cells were defined by terminal-deoxynucleotidyltranferase (TdT)-mediated deoxyuracil triphosphate (dUTP) nick-end labeling (TUNEL) using the ApopTag Plus Peroxidase in situ apoptosis detection kit (Merck Millipore, Darmstadt, Germany). The assay was performed in accordance with instructions of manufacturer, with minor modifications. After the color reaction with DAB (3,3′diaminobenzidine) apoptotic cells were observed brown, while counter staining was managed using methyl green to observe living cells. Apoptotic cells were detected by standard light microscopy, live and death cells were counted by two independent observersin a minimum of 5 frames.

### Statistical analysis

Survival curves were plotted according to the Kaplan-Meier method, the log-rank test being used to determine significant differences with respect to expressions. Correlations were determined by Spearman correlation tests. Statistical analyses were performed using the SPSS package (Version 16.0; SPSS Inc., Chicago, IL, USA).

## Abbreviations

TRF-2: Telomere repeat-binding factor-2
POT1: Protection of telomeres protein 1
TERT: Telomerase reverse transcriptase
Bcl-xL: B-cell lymphoma-extra-large
ATM: Ataxia Telangiectasia Mutated kinase
NHEJ: Non-homologous end joining
ROS: Reactive-oxygen-species.
